# Pakistan’s first medicine price deregulation policy: assessing its impact on prices, affordability, and availability of oral anti-diabetic medicines in private pharmacies

**DOI:** 10.3389/fphar.2025.1627735

**Published:** 2025-07-16

**Authors:** Amna Saeed, Sundus Shukar, Najwa Ali Yasin, Caijun Yang, Minghuan Jiang, Muhammad Majid Aziz, Haris Zahoor, Muhammad Sunnan-Ud-Din, Yu Fang, Zaheer-Ud-Din Babar

**Affiliations:** ^1^ Department of Pharmacy Administration and Clinical Pharmacy, School of Pharmacy, Xi’an Jiaotong University, Xi’an, Shaanxi, China; ^2^ Center for Drug Safety and Policy Research, Xi’an Jiaotong University, Xi’an, Shaanxi, China; ^3^ Department of Pharmacy Practice, Faculty of Pharmacy, Bahauddin Zakariya University, Multan, Pakistan; ^4^ Faculty of Pharmaceutical Sciences, Riphah International University, Islamabad, Pakistan; ^5^ College of Pharmacy, Qatar University, Doha, Qatar

**Keywords:** medicine price-deregulation, anti-diabetic medicines, access to medicines, pharmaceutical policy, national essential medicines list

## Abstract

**Introduction:**

Pakistan’s highest diabetes prevalence necessitates equitable access to anti-diabetic medicines. This study evaluated the access to Oral antidiabetics (OADs) and the effect of Pakistan’s recently launched price deregulation policy—applicable to medicines not included on the National Essential Medicines List (non-NEML)—on their prices and affordability by comparing NEML and non-NEML OADs.

**Methods:**

A WHO/HAI methodology-based survey in 30 private pharmacies across six regions gathered prices and availability data of 30 OADs, including the Lowest Price Generic (LPG), Highest Price Generic (HPG), and originator brand (OB). These selected OADs consisted of 11 products from NEML and 19 non-NEML products, comprising 17 single-active ingredient and 13 multi-active ingredient formulations. Published and surveyed retail prices of OADs (in Pakistani Rupees, PKR) before and after deregulation were compared, and the policy’s effect was determined by difference-in-differences (DiD) analysis. Affordability for the lowest-paid employee and medicine availability in percentages were calculated.

**Results:**

The DiD analysis revealed that the unit prices of OADs were significantly increased by PKR 15.08 (OB), PKR 5.89 (HPG), and PKR 2.81 (LPG) (*p* < 0.05) within just 6 months of the policy’s introduction. Medicines listed on the NEML remained consistently cheaper than non-NEML, with differences of −30.20 for OBs, −9.83 for HPGs, and −7.51 for LPGs in PKR (*p* < 0.001). As per DiD interaction terms (NEML enlistment status × deregulation), a greater increase in prices of non-NEML OBs was observed compared to NEML counterparts (PKR −10.85, *p* ≈ 0.05), while differences observed for LPGs (PKR 0.77, *p* = 0.73) and HPGs (PKR -0.20, *p* = 0.95) were insignificant. Prices of both single and multi-active ingredient formulations also increased significantly (*p* < 0.05). Although most OADs had fair availability from 47% to 97% after deregulation, seven out of 30 OADs remained unaffordable at both time points, and the overall affordability declined significantly post-deregulation (*p* < 0.05).

**Conclusion:**

The study revealed significant price escalations for most OADs, particularly those not enlisted on NEML, highlighting access challenges for diabetic patients and necessitating targeted policy reforms that address key market-related factors to ensure equitable access to OADs.

## 1 Introduction

Diabetes mellitus is one of the top ten causes of death globally and has emerged as the fastest-growing global health concern (Hossain et al., 2024; World Health Organization, 2024). The World Health Organization (WHO) reports that since 2000, the number of diabetes-related deaths worldwide has increased by 95%. In lower-middle-income countries (LMICs), diabetes is becoming a more common cause of mortality. Since 2000, the death toll from this disease has more than doubled, and it has risen from the 14th to the 8th position (World Health Organization, 2024). According to the International Diabetes Federation, 10.5% of the world’s adult population currently has diabetes, a figure projected to rise to 11.2% by 2045, with nearly 90% of these cases being type 2 diabetes (Kaiser et al., 2018; Sun et al., 2022). Alarmingly, Pakistan has the highest global prevalence of diabetes, with 30.8% of its population affected, and this number is expected to increase to 33.6% by 2045 (Sun et al., 2022).

The high prevalence of diabetes demands an urgent need for access to affordable and effective treatment options. Oral antidiabetics (OADs), crucial for maintaining glycemic control and minimizing complications associated with the disease, are the backbone of diabetes therapy, particularly type 2 diabetes (Qaseem et al., 2017; Yu et al., 2022). Notably, several key OADs are part of the 23rd WHO Model Essential Medicine List (EML) and are also part of Pakistan’s National Essential Medicines List (NEML) 2023 (World Health Organization, 2023; DRA, 2023). Essential medicines are defined as those that meet the priority healthcare needs of a population, warranting their availability in functioning health systems at all times and at affordable prices (World Health Organization, 2023; Wirtz et al., 2017). Furthermore, access to essential medicines is also recognized as a critical component of the Sustainable Development Goals (SDG) by the United Nations (UN), emphasizing the significance of equitable healthcare access for all (United Nations, 2015). In addition to essential medicines, many other OADs, including newer drugs and fixed-dose combinations (FDCs), are frequently prescribed for diabetic patients, especially those with comorbidities, due to their enhanced clinical benefits, such as better glycemic control and improved adherence (Kalra et al., 2014; Bangalore et al., 2007; Ramzan et al., 2019). Thus, ensuring access to these “non-essential” diabetes medicines is also important, especially in countries with high prevalence of diabetes.

Access to diabetes medicines has been reported to be poor in LMICs, where effective disease management is hindered by significant barriers, such as high prices, inadequate accessibility, and deficient healthcare infrastructure (Khatib et al., 2016; Castillo-Laborde et al., 2022; Kibirige et al., 2022; Babar et al., 2019; Butt et al., 2024). Like many other LMICs, Pakistan offers affordable public-sector medications. However, patients frequently turn to private-sector pharmacies to get necessary prescriptions because of their limited availability in the public sector (Saeed et al., 2019; Saeed et al., 2022; Saleem et al., 2021; Saeed et al., 2021; Bibi et al., 2022). This reliance on the private sector led to high out-of-pocket (OOP) expenditure in Pakistan, accounting for 54% of total health spending, underscoring the country’s poor financial protection for healthcare expenditures (Khalid et al., 2021). In February 2024, the federal government of Pakistan approved the proposal for deregulation of medicine prices, permitting market-driven pricing for non-essential medications, including numerous OADs. One of the main goals of this new policy was to reduce medicine prices and enhance their availability through increased market competition (Tribune, 2024). Previously, the Maximum Retail Prices (MRPs) of all medicines in Pakistan were strictly regulated, making the recent shift to a more flexible pricing policy a significant change in the country’s pharmaceutical landscape (Saeed et al., 2020). While price deregulation increases the chances of profitability for manufacturers by allowing them to set the medicine prices, it also poses a greater risk of higher prices, particularly for medicines in markets with poor competition, which could compromise equitable access to medicines. It is important to highlight that prior research has shown that LMICs like Pakistan often lack a systematic approach to medicine price regulations. This underlines the lack of evidence-based interventions and the inability to implement policy measures despite the available literature on medicine pricing issues (Babar, 2022; Babar, 2024; Vogler et al., 2024).

In light of this, monitoring medicine prices, particularly in private pharmacies, where patients pay OOP, is crucial to assess the impact of the recent policy shift. While some studies have indicated that certain OADs are affordable in Pakistan, there is limited data on newer drugs and fixed-dose combinations (FDCs), many of which are frequently prescribed yet are not included on the NEML (Babar et al., 2019; Saeed et al., 2019; Saeed et al., 2022; Butt et al., 2023). Therefore, this study aims to evaluate access to both NEML and non-NEML OADs in Pakistan, by analyzing their prices, affordability, and availability after deregulation, while also comparing the effects of price deregulation on single-active-ingredient (SAI) formulations and FDCs.

## 2 Materials and methods

### 2.1 Study design and region

This was a cross-sectional survey-based study conducted using a variant of WHO/Health Action International (HAI) methodology (Health Action International, 2018). A total of 30 OADs were selected for the survey, with different strengths of the same medicine considered as separate products. According to the standard WHO/HAI methodology, the survey region must be split into six areas according to the government-defined levels of administration (e.g., cities, and districts). In this study, six survey areas (cities) across Pakistan were selected for the survey, ensuring representation from each province and the federal capital (Supplementary Figure S1). These included Islamabad (the federal capital), Lahore (the provincial capital of Punjab), Faisalabad (a major city in Punjab), Peshawar (the provincial capital of Khyber Pakhtunkhwa), Karachi (the provincial capital of Sindh), and Quetta (the provincial capital of Baluchistan). Punjab, being the most populous province, was represented by two regions. This selection includes at least one city from each province and a separate inclusion of the federal capital.

### 2.2 Sampling of survey units

According to the standard WHO/HAI methodology, the public sector medicine outlets serve as the sample’s reference point, and other kinds of medicine outlets are selected based on how close they are to these outlets. A list of all public sector medicine outlets was obtained and one biggest public sector hospitals in each survey area was selected as a primary survey anchor (Health Action International, 2018). Subsequently, 4 public sector hospitals were chosen within 3 h of travel from the main hospital. These hospitals were not part of the data collection but served as reference points for selecting nearby private pharmacies. 5 private retail pharmacies were selected, including one pharmacy within 10-km radius of each hospital. The current study applied this sampling strategy consistently across all six survey areas, focusing on data collection from registered private retail pharmacies only. This approach was taken for two main reasons: (1) in Pakistan, the medicines are provided free of charge to the patients in public sector hospital pharmacies, and (2) the availability of medicines in public sector pharmacies is often limited, leading patients to depend mainly on private retail pharmacies, where they pay OOP to obtain these medications (Khalid et al., 2021; Saeed et al., 2020). A similar focused survey in private pharmacies using an adapted WHO/HAI methodology has been conducted previously (Saleem et al., 2021). Finally, a total of 30 retail pharmacies, 5 in each survey area, were selected across the country for this survey.

### 2.3 Selection of medicines to be surveyed

A total of 30 OADs were systematically selected for inclusion in the survey. First, a comprehensive list of all registered OADs was prepared using publicly available online sources, including drug information system and PharmaGuide (PharmaGuide, 2024; Drug Information System, 2024). This list consisted of a total of 903 brands, comprising 97 generic products with various strengths and dosage forms. The annual procurement lists of three tertiary care hospitals in the survey regions were considered to include the frequently procured OADs (n = 18). All strengths of oral diabetes medicines with therapeutic alternatives from NEML and WHO EML were included in the list (n = 17), and literature was searched to enrich the list further (Butt et al., 2023). A preliminary list of 74 commonly used OADs was prepared following these steps. Finally, six endocrinology consultant physicians and two general physicians working in major hospitals from the survey areas were invited to rate the 74 OADs based on the frequency of prescriptions in their practice setting. They assigned a score from 1-3 to each drug, where: 1 denoted “rarely prescribed”, 2 indicated “occasionally prescribed”, and 3 represented “frequently prescribed”. The top 30 medicines with higher scores were selected for the survey.

The final list included both the essential OADs enlisted in the NEML 2023 (n = 11), and 19 non-NEML products. Of these selected medicines 17 had SAIs and 13 were FDCs. Multiple strengths of 5 frequently used medicines-Metformin, Glimepiride, Empagliflozin, Metformin/Sitagliptin, and Metformin/Glimepiride-were also included. Glibenclamide 5mg, the only antidiabetic medicine included in the global core list of medicines specified in the WHO/HAI survey manual was included (Health Action International, 2018) (See Supplementary Table S1).

### 2.4 Data collection

The data were collected by trained data collectors, for the Lowest Price Generic (LPG), Highest Price Generic (HPG), and Originator Brand (OB) of the surveyed medicines, using a standardized data collection form during June-July 2024. Where OB is the innovator brand that first received market authorization, HPG is the generic product of selected medicine with the highest price, and LPG is generic with the lowest price available at a survey outlet. Availability and maximum retail prices were noted after physically checking the stock. If a facility had only one generic product of medicine, it was categorized as LPG and HPG for price comparison analysis. A similar approach has been followed in another study (Satheesh et al., 2020). To enable a comparison between current (post-deregulation) prices of all available OAD products in the Pakistani market and their pre-deregulation prices, historical price data were obtained for each product found in the post-deregulation survey, from the PharmaGuide, 31st edition (2024). This edition of the Pharma Guide provides the last drug regulatory authority of Pakistan (DRAP)-regulated prices as of December 1, 2023, immediately before the deregulation of non-NEML medicines (PharmaGuide, 2024). Generally recognized as a reliable public source for medicine pricing, the PharmaGuide has been referenced in several studies for establishing baseline prices, making it a dependable resource for retrospective analyses (Rafi et al., 2021; Kamran et al., 2020; Ayub et al., 2022).

### 2.5 Data analysis

The data was analyzed by using Microsoft Excel and Stata (version 15; StataCorp LLC, College Station, TX, United States).

#### 2.5.1 Prices

For analysis of prices, the unit prices of each surveyed OADs, defined as price per capsule or tablet, were considered. The unit prices were calculated using the following equation:

$$Unit\;price=\frac{Price\;of\;package\;of\;medicine\;found}{Pack\;size\;of\;medicine\;found}$$

The prices were reported as Median Unit Prices (MUPs) categorized by medicine, price type (LPG, HPG, OB), NEML status (NEML and non-NEML medicines), and formulation type (SAI and FDC). The three price types were compared across two primary grouping variables, NEML status, and formulation type, before and after the deregulation policy using the Wilcoxon rank-sum test. Statistical significance was determined at a *p*-value threshold of 0.05. Observations with missing price data were excluded to ensure accuracy in comparisons. This comparative analysis was performed to set a foundation for the subsequent Difference-in-Differences (DiD) analysis to assess the impact of deregulation policy.

#### 2.5.2 Impact of price deregulation on medicine prices

DiD analysis, a quasi-experimental approach, was applied to evaluate the effects of price deregulation on medicine prices in Pakistan. It is a widely recognized method to measure the impact of health policies (Zhang et al., 2022b; Teng et al., 2024). The analysis compared price changes in NEML medicines (control group/unexposed to deregulation) and non-NEML medicines (treatment group/exposed to deregulation policy), before and after the deregulation policy’s implementation. The data included unit prices of both NEML and Non-NEML OADs across three pricing categories (LPG, HPG, and IB), pre and post price deregulation policy. In the DiD model, we specified three regression equations.

$$\text{For LPG}:LPG_{price}=\beta_{o}+\beta_{1}Post_{deregulation}+\beta_{2}NEML_{status}+\beta_{3}\left(Post_{deregulation}\times NEML_{status}\right)+\epsilon$$

$$\text{For HPG}:HPG_{price}=\alpha_{o}+\alpha_{1}Post_{deregulation}+\alpha_{2}NEML_{status}+\alpha_{3}\left(Post_{deregulation}\times NEML_{status}\right)+\epsilon$$

$$\text{For IB}:IB_{price}=\gamma_{o}+\gamma_{1}Post_{deregulation}+\gamma_{2}NEML_{status}+\gamma_{3}\left(Post_{deregulation}\times NEML_{status}\right)+\epsilon$$

In each equation, $\beta_{o}$ , $\alpha_{o}$ , and $\gamma_{o}$ represent the intercepts or base price levels; $\beta_{1},\alpha_{1}$ , and $\gamma_{1}$ capture the effect of the deregulation policy on unit prices; $\beta_{2},\alpha_{2}$ , and $\gamma_{2}$ indicate price differences based on NEML status; and, $\beta_{3},\alpha_{3},$ and $\gamma_{3}$ represent the interaction term, estimating the difference-in-differences effect, or the relative price change of Non-NEML medicines compared to NEML medicines post-deregulation. To guarantee consistency, robust standard errors were fitted to DiD regression models. The robustness of the model was validated by sensitivity studies, which included a placebo test.

The formulation type variable was not included in the DiD analysis because all of the FDCs were non-NEML i.e., the exposed group. So, the price changes across FDCs and SAIs after deregulation were assessed separately through linear regression models. To analyze whether the price changes varied between these groups, interaction terms were also included, as per the following general model:

$$Y_{ij}=\beta_{o}+\beta_{1}{Period}_{j}+\beta_{2}{FDC}_{i}+\beta_{3}\left({Period}_{j}\times {FDC}_{i}\right)+\epsilon$$

Where, $Y_{ij}$ is the unit price of medicine i in period j (e.g., LPG, HPG, or OB prices). The constant term $\beta_{o}$ reflects the baseline average price for SAI medicines in the pre-deregulation period. $\beta_{1}$ captures the price change for SAI medicines between pre- and post-deregulation periods, while $\beta_{2}$ represents the baseline price difference between FDCs and SAI medicines. The interaction term coefficient $\beta_{3}$ estimates the differential impact of deregulation on FDCs compared to SAI medicines, with ϵ as the error term.

The relationship between the number of registered brands and pre-post prices (including LPG, HPG, and OB) was also evaluated through linear regression models.

#### 2.5.3 Affordability

The affordability of surveyed OADs was calculated in terms of the number of days’ wages (NDWs) required for a lowest-paid government employee to afford the standard treatment course. The defined daily dose (DDD) of each OAD, which is the “assumed average maintenance dose per day for a drug used for its main indication in adults,” was used as a standard dose unit of measurement in the analysis of affordability for standard treatment of each medication (Deressa et al., 2024). The monthly treatment cost and NDWs were calculated by using the following equations:

Treatment course cost=Number of unit dose required for DDD of OHGAx Median unit price of OHGAxdays of treatment

$$Number\;of\;days^{\prime }wages\;required=\;\frac{Treatment\;course\;cost\;\left(local\;currency\right)}{Per\;day\;wage\;of\;lowest\;paid\;government\;worker\;\left(local\;currency\right)}$$

The salary taken for the analysis was 32000PKR per month, i.e., 1066 PKR or 3.76 USD per day (with effect from July 2023). Notably, this threshold closely aligns with the World Bank’s lower-middle-income poverty line of $3.65/day (2017 PPP)–a metric encompassing 39.4% of Pakistan’s population in fiscal year 2023–24 (World Bank, 2023). According to the standard WHO/HAI methodology, the medicine is considered affordable only if the lowest-paid unskilled government employee spends less than 1 day’s wage to get the standard treatment from that medicine (Health Action International, 2018). The Wilcoxon signed-rank test was used to compare the affordability of surveyed drugs, before and after the deregulation policy, by stratifying the data into two categorical variables—NEML status and formulation type. Descriptive statistics were computed for each variable.

#### 2.5.4 Availability

Availability was reported in two categories OBs and generics (LPG/HPG). It was calculated as the percentage of a particular medicine available at each facility on the day of data collection, using the following equation:

$$Availability\left(\%\right)=\frac{Number\;of\;facilities\;with\;the\;medicine\;available}{Total\;number\;of\;facilities\;surveyed}x100$$

Only post-deregulation availability data were considered in the analysis. The percentage availability was categorized as follows: absent, indicating that 0% of facilities surveyed had the enlisted medicines at the time of the survey; low, indicating that less than 50% of facilities had the surveyed enlisted medicines; fairly high, indicating that 50%–80% of facilities had the surveyed enlisted medicines; high, indicating that more than 80% of facilities had the surveyed enlisted medicines, with them being found in most of the facilities (Saeed et al., 2019). To compare the availability of OADs, we conducted a series of two-sample t-tests with equal variances, comparing mean availability across different groups categorized by NEML status, price category, and formulation type.

## 3 Results

### 3.1 Medicines prices before and after the price deregulation policy

Overall, the prices of OADs in Pakistan exhibited a noticeable increase of up to 296% after the launch of the price deregulation policy.

#### 3.1.1 Medicine-specific analysis of prices

The top five OADs with the highest increase in MUPs were Rosiglitazone 4 mg Tabs (LPG: 296.23%), Glipizide 5 mg Tabs (LPG: 174.05%), Gliclazide 80 mg Tabs (LPG: 63.65%; HPG: 87.76%), Glimepiride/Pioglitazone 4mg/15 mg Tabs (LPG: 88.78%; HPG: 131.02%), and Vildagliptin 50 mg Tabs (OB: 70.04%) (See Table 1; Supplementary Table S1).

**TABLE 1:** Median unit prices (MUPs) by medicine type before and after price deregulation.

Sr. no.	Generic name, strength drug form	Lowest price generics			Highest price generics			Originator brands		
		MUP- pre. (PKR)	MUP- post. (PKR)	Change (%)	MUP- pre. (PKR)	MUP- post. (PKR)	Change (%)	MUP-pre (PKR)	MUP-post (PKR)	Change (%)
1	Sitagliptin 100 mg Tabs	49.80	53.43	7.29	52.13	54.45	4.46	110.70	111.00	0.27
2	Vildagliptin 50 mg Tabs	24.00	28.57	19.04	30.39	38.04	25.17	79.17	134.62	70.04
3	Repaglinide 2 mg Tabs	11.48	14.41	25.53				59.80	59.80	0.00
4	Metformin 500 mg Tabs	3.26	3.26	0.00		3.20		3.56	3.72	4.49
5	Metformin 1 g Tabs	3.62	4.02	11.05	4.02	4.02	0.00	6.19	7.07	14.22
6	Empagliflozin 25 mg Tabs	34.29	32.14	−6.27	40.00	44.78	11.95			
7	Empagliflozin 10 mg Tabs	24.29	31.22	28.51	24.29	35.00	44.09			
8	Dapagliflozin 5 mg Tabs	37.36	37.36	0.00	37.49	37.49	0.00			
9	Glipizide 5 mg Tabs	3.43	9.4	174.05	3.43	3.5	2.04			
10	Gliclazide 80 mg Tabs	13.08	21.405	63.65	13.56	25.46	87.76	13.56	13.56	0.00
11	Glimepiride 1 mg Tabs	8.00	10.17	27.13	8.75	11.02	25.89	8.62	10.34	19.95
12	Glimepiride 2 mg Tabs	13.57	15.00	10.58	14.53	20.40	40.40	20.50	20.50	0.00
13	Glimepiride 3 mg Tabs	19.00	20.00	5.26	19.00	23.90	25.79	36.23	46.20	27.52
14	Glimepiride 4 mg Tabs	19.88	28.68	44.28	28.00	37.07	32.39	41.00	41.01	0.02
15	Glibenclamide 5 mg Tabs	3.20	3.31	3.44	3.43	4.12	20.12			
16	Pioglitazone 15 mg Tabs	21.29	25.71	20.76	29.64	29.64	0.00			
17	Rosiglitazone 4 mg Tabs	2.65	10.50	296.23						
18	metformin/sitagliptin 1 g/50 mg Tabs	33.71	37.50	11.24	34.14	41.61	21.87	71.43	111.00	55.40
19	metformin/sitagliptin 500 mg/50 mg Tabs	34.15	34.50	1.03	37.43	41.00	9.54	71.43	96.87	35.62
20	Vildagliptin/metformin 50 mg/1 g Tabs	38.57	38.57	0.00	38.93	39.29	0.92	74.15	103.33	39.35
21	Metformin/Pioglitazone 500 mg/15 mg Tabs	14.04	14.19	1.07	14.04	15.71	11.89			
22	Metformin/Rosiglitazone 1 g/2 mg Tabs	12.30	13.81	12.28	9.68	9.68	0.00			
23	Glipizide/Metformin 5 mg/500 mg Tabs	12.60	13.81	9.60						
24	Glimepiride/Pioglitazone 4 mg/15 mg Tabs	20.64	38.97	88.78	18.86	43.57	131.02			
25	Metformin/Glibenclamide 500 mg/5 mg Tabs	5.68	6.65	17.08				5.69	7.970	40.07
26	Metformin/Glimepiride 500 mg/2 mg Tabs	17.57	18.76	6.77	17.27	20.23	17.14	37.80	37.80	0.00
27	Metformin/Glimepiride 500 mg/1 mg Tabs	10.50	12.00	14.29	10.50	12.80	21.90	21.00	21.00	0.00
28	Ertugliflozin/Sitagliptin 15 mg/100 mg Tabs	45.86	49.64	8.24	45.86	55.36	20.72			
29	Empagliflozin/Linagliptin 10 mg/5 mg Tabs	30.78	33.75	9.65	37.70	41.43	9.89			
30	Metformin/ empagliflozin 1 g/10 mg Tabs	26.5	32.14	21.28	26.5	36.24	36.75			

#### 3.1.2 Prices of generics versus originator brands

**FIGURE 1:**
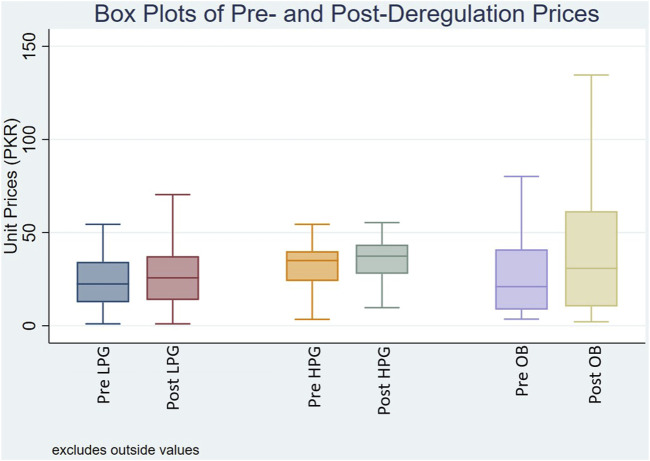
Box plot of median unit prices for oral antidiabetic medicines Pre- and Post-deregulation. Where, Pre/Post LPG: Unit prices of lowest price generics before and after deregulation policy; Pre/Post HPG: Unit prices of highest price generics before and after deregulation policy; Pre/Post OB: Unit prices of originator brands before and after deregulation policy.

In Figure 1, the box plot illustrates the distribution of MUPs for OADs categorized by price types: LPG, HPG, and OB, before and after the deregulation policy. The MUPs for surveyed medicines showed an upward trend across all price categories. For LPGs, the MUP rose moderately from PKR 22.43 to PKR 25.71. HPGs showed an increase in MUPs from PKR 35 to PKR 37.36. For OBs, the MUP showed a marked rise from PKR 21 pre-deregulation to PKR 30.80 post-deregulation. A significant change in price distribution reflected by the IQRs widening following deregulation underscores a wider dispersion of prices across all price types. Supplementary Table S2 provides the summary statistics of MUPs for OADs by price category pre- and post-deregulation.

#### 3.1.3 Analysis of prices by NEML enlistment status

Overall, the NEML medicines were priced lower than non-NEML medicines both before and after deregulation (*p* < 0.05). Details on the medians and statistical comparison of MUPs of OADs by NEML status, price, and formulation types, before and after deregulation, can be found in Supplementary Table S3. Interestingly, the price trend for NEML medicines was HPG > LPG > OB, whereas for non-NEML medicines the order reversed to OB > HPG > LPG. The MUPs of non-NEML OBs remained stable at PKR 59.80 post-deregulation, while MUPs of LPGs increased from PKR 26.5 to PKR 30.36, and HPGs rose from PKR 37.7 to PKR 39.29. In contrast, for NEML OBs, the MUPs increased modestly from PKR 13.56 PKR to PKR 15.51, whereas LPGs increased from PKR 18 PKR to PKR 20.39, and HPGs rose from PKR 24.29 to PKR 35. Notably, the average number of products registered for NEML and non-NEML medicines was 37 and 9, respectively.

#### 3.1.4 Impact of price deregulation policy on medicine prices: Results of DiD analysis

The results of DiD analysis confirmed statistically significant price increases for all categories following deregulation (See Table 2). The biggest hike was observed in OBs (PKR 15.05, *p* = 0.004), followed by HPGs (PKR 5.97, *p* = 0.02) and LPGs (PKR 2.91, *p* = 0.01), suggesting that all prices were increased for these categories within 6 months of the policy’s introduction. It also confirmed that the NEML medications were consistently less expensive than non-NEML medications. The price differences between NEML and non-NEML medicines (in PKR) for OBs, HPGs, and LPGs were −30.20 (p < 0.001), −9.83 (*p* < 0.001), and −7.51 (*p* < 0.001), respectively, highlighting the protective function of NEML inclusion in preserving affordability. The interaction term (Period × NEML status), expressing the differential price effect of deregulation between NEML and non-NEML medicines, revealed no statistically significant difference in costs between NEML and non-NEML medications for LPGs (β = 0.77, *p* = 0.35) and HPGs (β = −0.20, *p* = 0.95) in PKR, after controlling for baseline differences. OBs, on the other hand, a somewhat greater and a marginally significant negative interaction was noted (β = −10.85, *p* = 0.05), indicating that NEML OB prices rose 10.85 units less after deregulation than non-NEML OB prices. These results suggest that, in contrast to their NEML counterparts, non-NEML OB prices were more significantly raised by the price deregulation, although the effects were less noticeable for LPGs and HPGs. Notably, the lower R-squared values suggest other unmeasured and important factors might have affected the medicine prices.

**TABLE 2:** Impact of price deregulation on unit prices of OADs: Difference-in-Differences regression analysis.

Variables	LPG prices (PKR)	HPG prices (PKR)	OB prices (PKR)
	Coef. (Robust SE)	Coef. (Robust SE)	Coef. (Robust SE)
Period (Post vs. Pre-deregulation)	2.91 (1.15) **	5.97 (2.55) **	15.05 (5.23) ***
NEML Status (NEML vs., non NEML)	−7.51 (1.14) ***	−9.83 (1.72) ***	−30.20 (3.07) ***
DiD Interaction Term (Period × NEML status)	0.77 (2.22)	−0.20 (3.28)	−10.85 (5.52)
Constant	26.72 (0.77) ***	35.63 (1.04) ***	49.61 (2.83) ***
Observations	1,028	460	518
F-statistic	27.59***	16.16***	60.02***
R-squared	0.05	0.09	0.27

Where, Dependent Variables: Unit prices of Lowest price generics (LPG), Highest price generics (HPGs), and Originator Brands (OB) pre- and post-deregulation policy.

Significance levels: *p* < 0.05*, *p* < 0.01**, *p* < 0.001***.

#### 3.1.5 Prices of single versus multi-active ingredient formulations

Upon stratification by formulation type, the Wilcoxon rank-sum test revealed the prices of LPG and OB of FDCs were significantly higher than those of SAI medicines, while for HPG FDCs the difference was not significant (*p* < 0.01), both before and after deregulation (See Supplementary Table S3). This trend was further confirmed by the regression results (Table 3), where the baseline unit prices of FDCs were considerably higher than SAI medicines with a difference of PKR 3.05 (*p* = 0.0009) for LPG, PKR 2.88 (*p* = 0.09: Not significant), and PKR 14.66 (*p* < 0.001). The findings also revealed that the prices of both SAI medicines and FDCs increased significantly for all price types (LPG, HPG, and OB) after deregulation. The interaction terms between formulation type and period (pre-post-deregulation) indicated no significant differential impact of deregulation on FDCs compared to SAI medicines across all price types.

**TABLE 3:** Effect of price deregulation on unit prices of OADs (PKR) by formulation type: regression analysis results.

Variables	LPG prices (PKR)	HPG prices (PKR)	OB prices (PKR)
	Coef. (Robust SE)	Coef. (Robust SE)	Coef. (Robust SE)
Period (Pre and Post-deregulation policy)	3.83 (1.61) **	4.77 (1.98) **	6.67 (3.34) ***
FDC Baseline Difference	3.06 (1.16) ***	2.89 (1.72)	14.67 (3.72) ***
DiD Interaction Term (Period × Formulation type)	−1.18 (2.00)	2.24 (3.77)	10.81 (7.23)
Constant	22.24 (0.82) ***	30.39 (1.26) ***	29.31 (1.96) ***
Observations	1,028	460	518
F-statistic	7.78***	4.06***	12.89***
R-squared	0.01	0.03	0.08

Where, Lowest price generics: LPG; Highest price generics: HPG; and Originator Brands: OB. Significance levels: *p* < 0.05*, *p* < 0.01**, *p* < 0.001***.

#### 3.1.6 Medicine prices and market competition

The findings from the linear regression models revealed a significantly negative association between prices and the number of registered brands (reflecting market competition), both before and after deregulation (See Supplementary Table S4). For LPGs, the addition of each registered brand was associated with a decrease in unit price by PKR 0.26 pre-deregulation and PKR 0.20 post-deregulation (*p* < 0.001). For HPGs, the unit prices were reduced by PKR 0.33 and PKR 0.39 pre and post-deregulation (*p* < 0.001). OBs presented a relatively greater reduction in unit prices PKR 0.52 (*p* < 0.001) before deregulation and PKR 0.72 (*p* < 0.001) after deregulation.

### 3.2 Medicines affordability before and after the price deregulation policy

Overall, affordability of OADs in Pakistan significantly worsened post-deregulation, as the Wilcoxon signed-rank test revealed a significant increase in median NDWs from 0.67 to 0.80 for LPG, 0.79 to 1.04 for HPG, and 1.16 to 1.24 for OBs (*p* < 0.01), indicating a decline in affordability by 19.40%, 31.65%, and 6.89% respectively, after deregulation (See Table 4).

**TABLE 4:** Affordability of medicines (no. of days’ wages) pre- and post-deregulation by NEML status, formulation type, and price categories.

Category	No. of days’ wages								
	Lowest price generics			Highest price generics			Originator brands		
	Pre	Post	Change (%)	Pre	Post	Change (%)	Pre	Post	Change (%)
By price type	0.67	0.80***	+19.40%	0.79	1.04***	+31.65%	1.16	1.24**	+6.89%
BY NEML Status									
NEML enlisted	0.45	0.57*	+26.67%	0.53	0.69**	+30.19%	0.53	0.57	+7.55%
Non-NEML	0.79	0.94***	+18.99%	1.01	1.17**	+15.84%	2.62	3.12**	+19.08%
BY Formulation Type									
SAI	0.53	0.60***	+13.21%	0.78	0.71**	+8.97%	0.79	0.86*	+8.86%
FDC	0.79	0.94**	+18.99%	0.87	1.16**	+33.33%	2.06	2.62	+27.18%

Significance levels: p < 0.05, **p < 0.01, ***p < 0.001.

NDWs: Number of days’ wages required to purchase a 30-day standard treatment; LPG: lowest price generic; HPG: highest price generic; OB: originator brand; NEML: national essential medicines list; PRE: Pre-deregulation period; POST: post deregulation period.

#### 3.2.1 Medicine-specific comparison of affordability

All available price types of 7 OADs–Sitagliptin 100 mg Tabs, Vildagliptin 50 mg Tabs, Empagliflozin 10 mg Tabs, Dapagliflozin 5 mg Tabs, Pioglitazone 15 mg Tabs, metformin/sitagliptin 500mg/50 mg Tabs, Vildagliptin/metformin 50 mg/1 g Tabs–remained consistently unaffordable both before and after deregulation. Interestingly, two of these medicines, Empagliflozin 10 mg Tabs and dapagliflozin 5 mg Tabs are included in both NEML and WHO EML 2023. Whereas, the generic products (LPG and HPG) of three OADs–metformin/sitagliptin 1g/50mg, Glimepiride/Pioglitazone 4mg/15mg, and Metformin/Glimepiride 500mg/2 mg–jumped from the affordable to the unaffordable category. Supplementary Table S5 provides the NDWs for each medicine before and after the price deregulation policy.

#### 3.2.2 Affordability of generics versus originator brands

The analysis revealed a significant increase in the NDWs for all price categories post-deregulation. The highest rise in median NDWs was noted for HPGs, increasing significantly by 31.65% from 0.79 to 1.04 (*p* < 0.001), bringing this category from affordable to unaffordable OADs. Although LPGs remained affordable but experienced a significant 19.40% rise in NDWs (*p* < 0.001). In contrast, the OBs exhibited minimal change in affordability by 6.89%, with median NDWs rising from 1.16 to 1.24 (*p* < 0.01). Although the change in the affordability of OBs was minimal, it is noteworthy that OBs remained unaffordable both before and after deregulation with median NDWs greater than 1 day.

#### 3.2.3 Affordability and NEML enlistment status

The affordability of NEML LPG and HPG significantly worsened by 26.67% (*p* < 0.05) and 30.19% (*p* < 0.01) respectively. In contrast, the rise in median NDWs was minimal (7.55%) and insignificant for NEML OBs. The non-NEML medicines observed more pronounced rises in median NDWs by 18.99% for LPGs (*p* < 0.001), 15.84% for HPGs (*p* < 0.01), and 19.08% for OBs (*p* < 0.01). Specifically, the median NDWs rose from 0.79 to 0.94 for LPGs, 1.01 to 1.17 for HPGs, and 2.62 to 3.12 for OBs, keeping the non-NEML HPGs and OBs unaffordable. This analysis highlights the need for policies to address affordability issues, particularly for non-NEML OADs.

#### 3.2.4 Affordability based on formulation type

The median NDWs for SAI medicines increased significantly by 13.21% (*p* < 0.001) for LPG, 8.97% (*p* < 0.01) for HPG, and 8.86% (*p* < 0.05) for OB. The median NDWs of FDCs also increased post-deregulation, by 18.99% (p < 0.01) for LPG, 33.33% (p < 0.01) for HPG, and 27.18% (*p* > 0.05) for OBs. All price types of SAIs remained affordable i.e., NDW<1, before and after deregulation. In the case of FDCs, the LPG remained affordable, the HPG shifted from affordable to the unaffordable category, and OBs remained unaffordable before and after deregulation (See Table 4).

### 3.3 Availability of oral antidiabetics post-deregulation

#### 3.3.1 Medicine-specific availability

About two-thirds of the surveyed OADs demonstrated moderate to high availability, with rates ranging from 47% to 97%. Metformin 500 mg and Sitagliptin 100 mg were the two most accessible OADs with 97% availability. However, almost half of the OADs (n = 16) fell below the WHO benchmark for availability, set at 80%.

#### 3.3.2 Availability of generics versus originator brands

As per the results of independent t-tests to compare mean availability across different medicine categories, the LPGs exhibited significantly higher mean availability (57.85%) compared to OBs (27.42%), with a statistically significant difference of 30.43% (p < 0.05) (See Table 5).

**TABLE 5:** Mean availability of Oral antidiabetics by price type, formulation type, and NEML status.

Category	Sub-category	Mean availability (%)	Difference	Standard error	t value	*p-value*
By medicine category	Generics	57.84	30.42**	8.06	3.75	0.00
	Originator Brands	27.42				
By formulation type	Single active ingredient	45.46	6.74	9.05	0.75	0.45
	Fixed dose combinations	38.71				
By NEML status	Non-NEML	41.41	−7.59	12.15	−0.6	0.53
	NEML	49				

NEML: National Essential Medicines List.

#### 3.3.3 Availability by NEML enlistment status

The NEML medicines were found to be more available than the non-NEML medicines with 49% and 41.41% availability, respectively. However, this difference of 7.59 was insignificant (*p* = 0.535) (See Table 5). Seven of the 30 surveyed OADs had low availability, including one NEML medicine, Glipizide 5 mg Tabs (23%) (See Supplementary Table S6).

#### 3.3.4 Availability by formulation type

SAI formulations had a mean availability of 45.46%, which was slightly higher than FDCs at 38.72%. However, the difference of 6.75% was not statistically significant (p = 0.459).

## 4 Discussion

The study offers a comprehensive evaluation of access to OADs and valuable insight into the immediate effects of the recently adopted medicine price deregulation policy on the prices and affordability of these medicines in the private sector of Pakistan. One of the primary purposes of this price deregulation policy was to foster market competition among non-NEML medicines manufacturers that could cause price reductions and better affordability for patients. Drug price deregulation policy has been adopted in several countries, and variable outcomes have been experienced for different medicine categories, most studies reported either minimal change or increases in medicine prices (Guan et al., 2019; Liu et al., 2017; Costa Font, 2016). In an LMIC like Pakistan, where the prevalence of diabetes is the highest and poor access to medicines is already a critical issue, this study gives critical evidence on current accessibility to antidiabetic medicines post-deregulation policy. It represents an effort to monitor the pharmaceutical market response to this significant policy change in Pakistan (Saeed et al., 2019; Saleem et al., 2016; Bhatti, 2024).

Our study has shown that the overall prices of OADs increased significantly especially for non-NEML medicines, thus aggravating the access challenges for diabetes patients. Notably, this upward trend was observed within just 6 months of the policy’s implementation, raising concerns about the potential for even greater increases over the long term due to the risk of market monopolization. Hence deregulation policy might have inadvertently led to price escalation rather than fostering competitive prices, especially for premium agents with less competitive environments. A study conducted in China found similar results and stated that price changes after policy implementation vary for drug categories (Zhang et al., 2022a). However, the availability for two-thirds of the surveyed OADs ranged from moderate to high, post-deregulation. This finding was partially consistent with the observations by Peruvian researchers who reported improved availability of certain generic medicines following deregulation (Costa Font, 2016).

### 4.1 Medicines prices before and after price deregulation policy

#### 4.1.1 Prices of generics versus originator brands

Overall, the HPGs were found to be the most expensive, followed by OBs and LPGs, at both time points. Certain generics had higher prices than OBs, possibly due to high generic entrants in the Pakistani market. The results align with the findings by Baber et al., who reported that OBs of some antidiabetic medicines were cheaper than their LPGs in some LMICs, contrary to the observation in a majority of the countries where OBs were priced higher (Babar et al., 2019).

After deregulation, the prices rose significantly across all categories—LPG, HPG, and OB. Similar findings on the effect of deregulation policy on different categories of medicines, with the majority facing price upsurges were reported by Guan et al. and Costa et al. in the Chinese and Peruvian markets (Guan et al., 2019; Costa Font, 2016). Our results highlighted that the policy might have broadly impacted the market, leading to overall price hikes and a possible rise of monopolistic practices among the manufacturers (Zhang and Bian, 2023).

#### 4.1.2 Medicine prices and NEML status

The deregulation policy targeted the non-NEML medicines, ideally, the prices of NEML medicines that were still under regulatory control should have stayed stable within this short period post-deregulation policy. However, the significant price surges of up to 296%, irrespective of their inclusion in the NEML 2023 translate into potentially inefficient implementation of the policy. Another contributing factor behind the lack of significant distinction between both NEML and non-NEML medicines could be the simultaneous price increments of NEML medicines e.g., prices of 146 essential drugs were increased in February 2024 alongside the launch of this deregulation policy for nonessential medicines (Azad, 2024). Nevertheless, the prices of NEML medicines remained lower than the non-NEML medicines at both time points. A multi-country study conducted by Bazarangi et al., also noted that essential medicines have significantly better accessibility than non-essential medicines (Bazargani et al., 2014). Notably, the OBs of NEML medicines were found to be high priced compared to generics while in the case non NEML medicines OBs were the most expensive. Reflecting the premium position of non-NEML OBs in the market. This disparity advocates for switching to the cheapest therapeutically equivalent alternative, which may lead to substantial cost savings (Abdullah et al., 2024).

#### 4.1.3 Impact of price deregulation on medicine prices-insights from DiD analysis

The DiD analysis also confirmed that the NEML-listed medicines, preserved lower prices compared to non-NEML medicines, indicating the protective role of NEML against price hikes. However, this protective effect did not entirely shield essential medicines from price escalation potentially stimulated by deregulation. Liu et al. also detected higher prices for essential medicines after the introduction of the deregulation policy, gravely impacting low-income populations (Liu et al., 2017). The non-NEML medicines especially for highly prevalent diseases like diabetes, have become increasingly unaffordable. This may lead to poor access to innovative treatment options, essential for specific groups of people, thus affecting healthcare equity. The effects of price deregulation differed depending on the type of medication i.e., LPG, HPG, and OB. While LPGs and HPGs did not show any obvious differential effects, the non-NEML OBs had a comparatively larger and significant price increase following the policy than their NEML counterparts. This implies that medications not listed on the NEML may have been disproportionately impacted by price deregulation, especially OBs, which are frequently more expensive and might be less vulnerable to competition. These findings highlight the significance of specific regulatory protections for expensive, non-NEML medications in order to avoid issues with affordability in the private sector.

#### 4.1.4 Medicine prices by formulation type

Increases in prices of both FDCs and SAI medicines were observed across all price categories (LPG, HPG, and OB) after the implementation of the deregulation policy. It was observed that all of the FDCs consistently commanded higher prices than SAI formulations across all price types before and after deregulation, reflecting their superior placement in the market (Hao et al., 2016).

#### 4.1.5 Medicine prices and market competition

The results highlight the significant impact of the number of registered brands representing the market competition on price drops among all categories, i.e., LPG, HPG, and OB. This effect was more prominent in OB prices, possibly due to higher price elasticity in this category. Nguyen et al. also noticed a 20% reduction in price for medicines with about three generic products in the market, and up to 80% price reductions for medicines with ten or more competitors (Nguyen et al., 2022). Chen Yina et al. also reported a price decline due to increasing market competition for drugs in China (Yina et al., 2023). The negative association between prices and market competition was more pronounced in post post-deregulation period, suggesting that the price deregulation policy might have augmented the role of market competition in bringing down the prices of OADs in the Pakistani market. The Peruvian market also showed lower prices for some medicines due to increased generic competition after the implementation of the deregulation policy (Costa Font, 2016). However, other factors related to market dynamics, including product-related, consumer-related, trading strategies, supply-related, and regulatory compliance, should also be considered to explore the impact of market competition along with deregulation on medicine prices (Borges dos Santos et al., 2019).

### 4.2 Medicines affordability before and after the price deregulation policy

The increase in the number of unaffordable medicines despite NEML’s protective status and the overall rise in NDWs for both NEML and non-NEML medicines highlight issues with the current pricing strategies. Strikingly, despite having more than 5 registered brands each, Empagliflozin 10 mg and Dapagliflozin, NEML medicines, remained among the top seven most unaffordable medicines. Whereas, gliflozins have proven cardiovascular benefits, in addition to effectively controlling blood glucose, by improving heart failure-related outcomes and preventing adverse events (Tornyos et al., 2022). However, Rahul et al. have also reported this particular class of OADs to be high priced and responsible for OOPs (Aggarwal et al., 2022). The reduction in affordability leads to prominent health disparities because patients might opt for alternatives with less efficacy or forego required medicines (Dzudie et al., 2020). A qualitative study of diabetic patients highlighted that patients have to face serious social and financial consequences, like buying cheaper medications and buying from the black market to support their costly treatment (Golder et al., 2021).

Overall, all three price types, i.e., LPG, HPG, and OBs, experienced significant rises in NDWs, worsening the affordability, post deregulation. The generics (LPGs and HPGs) had greater increments in NDWs compared to OBs, reflecting the possible monopolistic behaviour by local manufacturers (Zhang and Bian, 2023). While LPGs were found affordable, HPGs observed the greatest increment in NDWs, taking this category from affordable (pre-deregulation) to unaffordable (post-deregulation). Conversely, OBs experienced relatively minimal change post-deregulation; however, they remained largely unaffordable before and after deregulation, where the lowest-paid worker had to spend more than a day’s wage for monthly treatment with OBs.

The more pronounced decline in affordability observed among the non-NEML medicines compared to NEML medicines underscores the potential risks of blanket deregulation. The medicines for highly prevalent diseases like diabetes should be excluded from this new policy to safeguard patient affordability. A study conducted globally on different income levels for diabetic drugs further supported our finding, stating that availability and affordability were poor in LMICs (Chow et al., 2018).

Affordability declined for both formulation types, SAI and FDC. While all the SAIs and LPG of FDCs remained affordable, the HPG and OB of FDC were found to be unaffordable post-deregulation. The persistent unaffordability of FDC OBs highlights the need for policy intervention to alleviate the impact on diabetic patients who rely on these medicines (Kalra et al., 2020).

### 4.3 Availability

The post-deregulation analysis of availability for OADs in Pakistan revealed that a considerable proportion of these medicines had moderate to high availability, but a significant number of drugs were below the WHO 80% benchmark, highlighting access issues. This disparity in availability among various drugs underlines market preferences or supply chain efficiencies that might affect certain medicines. Some drugs, for example, Sitagliptin and Metformin, had high availability, probably due to a bigger market demand and a higher number of manufacturers/suppliers. Several other studies have also found Metformin 500 mg to have more than 80% availability in LMICs (Babar et al., 2019; Beran et al., 2018; Osuafor et al., 2021), reflecting not only its popularity but also the positive role of essential medicine lists. Conversely, the low availability of Glipizide, another essential medicine, may indicate reduced manufacturer attention, probably because of either lower demand, profitability, or regulatory challenges. Another study in Bangladesh reported that an inefficient drug supply chain was one suspected reason for low availability (Hakim et al., 2022).

Although the NEML OADs were slightly more available, there was no significant difference between the availability of NEML and non-NEML medicines, which is contrary to the findings by other researchers (Bazargani et al., 2014). This may also be credited to the local manufacturing capacity. The significantly higher availability of generics compared to OBs also underlines the high number of generic entrants in the Pakistani market. As the market is dominated by generics, the pricing of this category is crucial to guarantee access to these medicines for diabetic patients, especially those on low wages.

### 4.4 Implications

Although the deregulation policy has the potential to boost the pharmaceutical business and innovation in the country, its impact needs to be measured at a large scale, particularly for the poverty-stricken population of Pakistan (Baulch and McCulloch, 2002; Business Recorder, 2024). Our findings recommend that a blanket deregulation policy for non-NEML medicines, based on the supposition that it will uniformly reduce prices, seems to be flawed. A nuanced balance between demand, market competition, and the risk of monopolistic practices by manufacturers must be considered for effective regulation of medicine prices. A targeted approach to deregulation, informed by these market factors, is likely to yield better outcomes in ensuring affordability and accessibility. The regulators and policymakers should consider: i) Re-introducing price control policies for all antidiabetic medicines, so the affordability for diabetics is not compromised in a country like Pakistan with the highest global prevalence of diabetes; ii) Revising the section of medicines to treat diabetes in the NEML, considering the highest global prevalence of diabetes and its associated comorbidities like cardiovascular diseases; iii) Emphasizing the enforcement of NEML-based procurement; iv) employing targeted actions to implement generic prescribing, especially for non-NEML medicines; v) Conducting in-depth granular analysis, to comprehend pricing behaviors and manage regulatory procedures accordingly; vi) Studies should also be conducted to evaluate the treatment adherence as the high cost and compromised access lead to poor adherence, ultimately leading to increased morbidity and mortality (Saraiva et al., 2020).

### 4.5 Limitations

The present study had several limitations. First, the representativeness of findings may be limited by including only 30 OADs. However, the list of these medicines was meticulously prepared and represents the most frequently prescribed agents. This study is notably the first to include both FDCs and non-NEML medicines, providing novel insights into their access. Second, though the pre-deregulation prices were obtained from a reliable and widely recognized source for retrospective analysis, there may be variability compared to actual market prices before deregulation. This also restricted the availability analysis. Nevertheless, this study provides a baseline assessment of the deregulation policy’s effect and lays the foundation for future research and policy assessments. Third, the relatively low R-squared values in the DiD analysis highlighted that other important factors, such as pharmacy procurement prices, regional economic disparities, and supply chain issues, might have impacted the medicines’ retail prices. Subsequent studies should expand the dataset and include variables such as manufacturers’ policies, wholesale purchase data, regional economic indicators, and supply chain metrics to enhance the robustness of the model and to provide detailed analysis. Moreover, the DiD approach assumes that the treatment and control groups would have followed parallel trends in the absence of the intervention (Zhang et al., 2022b). Due to the use of a single pre-policy data point, we were unable to formally test this assumption. While non-NEML medicines exhibited higher prices than NEML medicines at both time points, this reflects differences in price levels, not trends, and thus the untestable assumption may affect the internal validity of our DiD estimates. This limitation should be considered when interpreting the policy effects. Additionally, macroeconomic variables such as inflation and exchange rate fluctuations were not controlled for in this analysis and may have independently influenced price trends. However, these factors would likely affect both NEML and non-NEML medicines similarly, and thus would not substantially bias our estimated policy impacts. Fourth, our 6-month post-deregulation evaluation may not reflect long-term market stabilization effects. While this timeframe provides crucial initial policy impact data, we strongly recommend future longitudinal studies to understand long-term market dynamics. In future research, it would also be important to account for the date of manufacture when collecting medicine price data, particularly in settings where government-regulated prices are frequently updated. This consideration can help ensure that price comparisons accurately reflect the policy’s impact on medicines manufactured under different pricing regimes.

## 5 Conclusion

This study highlights the critical challenges concerning access to the key OAD medicines posed by the price deregulation policy in Pakistan. A marked price hike was noted particularly among frequently prescribed non-NEML OB medicines, within just 6 months of the implementation of the policy. This trend has serious implications for diabetic patients, especially those with comorbidities and poor socioeconomic status. The study also highlights the protective but limited role of NEML against these price escalations. Although the availability of OADs was fair in the private sector, the affordability worsened significantly, reflecting the potential monopolistic practices by some manufacturers. These findings call for a policy review targeting a balance between the demand, market competition, and risk for monopolistic practices by manufacturers. Efforts should be made to ensure equitable access to medicines, especially for highly prevalent diseases like diabetes, on a priority basis.

## Data Availability

The raw data supporting the conclusions of this article will be made available by the authors, without undue reservation.
