# Supplementary material for: Pakistan’s first medicine price deregulation policy: assessing its impact on prices, affordability, and availability of oral anti-diabetic medicines in private pharmacies
Source: Front Pharmacol. 2025 Jul 16;16:1627735. doi: 10.3389/fphar.2025.1627735 (PMC12308141; doi:10.3389/fphar.2025.1627735)
Supplement: Supplementary file 1 [file Supplementaryfile1.docx]

**Supplementary material**

**
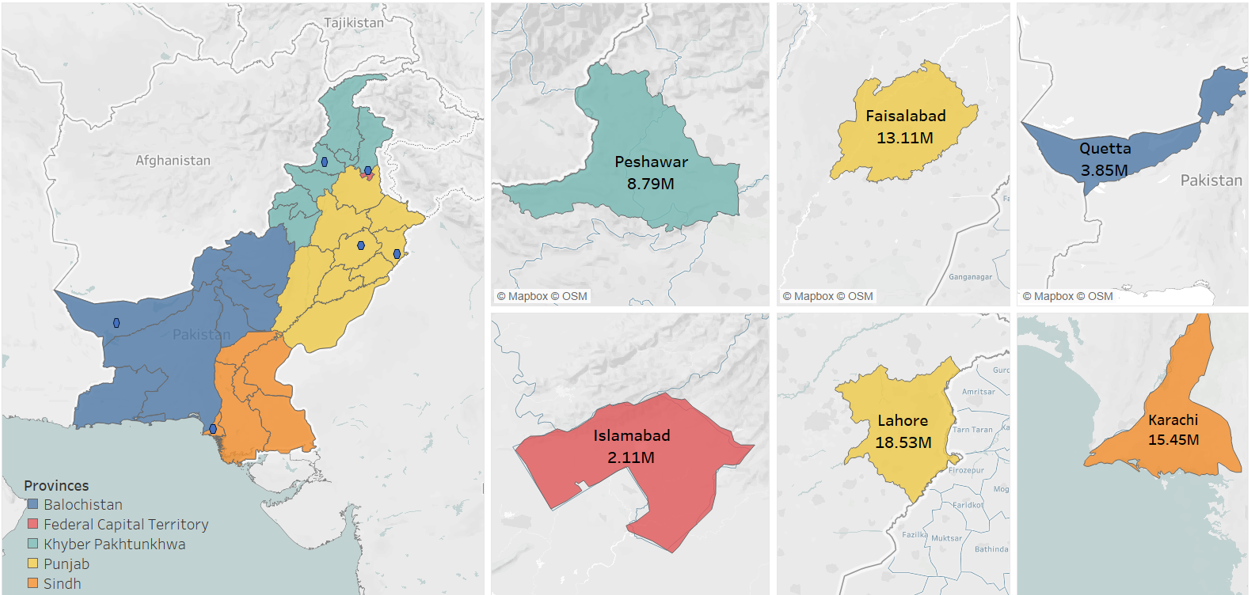
**

**Figure S1.** Survey regions and survey areas with estimated survey area population size.

**Table S1.** List of 30 oral antidiabetic medicines for the survey.

| Sr. no. | Pharmacological class | Generic name | Strength | Drug Form | NEML  2023 Enlistment | No. of brands in the market |
| --- | --- | --- | --- | --- | --- | --- |
| 1 | Dipeptidyl Peptidase 4 Inhibitors | Sitagliptin | 100mg | Tabs | No | 19 |
| 2 |  | Vildagliptin | 50mg | tabs | No | 9 |
| 3 | Meglitinides | Repaglinide | 2mg | Tabs | No | 8 |
| 4 | Non-Sulfonylureas | Metformin | 500mg | Tabs | Yes | 57 |
| 5 |  | Metformin | 1g | Tabs | No | 12 |
| 6 | Sodium-Glucose Cotransporter-2 Inhibitors | Empagliflozin | 25mg | Tabs | Yes | 12 |
| 7 |  | Empagliflozin | 10mg | Tabs | Yes | 12 |
| 8 |  | Dapagliflozin | 5mg | Tabs | Yes | 9 |
| 9 | Sulfonylureas | Glipizide | 5mg | Tabs | Yes | 5 |
| 10 |  | Gliclazide | 80mg |  | Yes | 32 |
| 11 |  | Glimepiride | 1mg | Tabs | Yes | 66 |
| 12 |  | Glimepiride | 2mg | Tabs | Yes | 40 |
| 13 |  | Glimepiride | 3mg | Tabs | Yes | 59 |
| 14 |  | Glimepiride | 4mg | Tabs | Yes | 64 |
| 15 |  | Glibenclamide | 5mg | Tabs | Yes | 30 |
| 16 | Thiazolidinediones | Pioglitazone | 15mg | Tabs | No | 37 |
| 17 |  | Rosiglitazone | 4mg | Tabs | No | 8 |
| 18 | fixed dose  combinations | Metformin/Sitagliptin | 1000mg/50mg | Tabs | No | 12 |
| 19 |  | Metformin/Sitagliptin | 500mg/50mg | Tabs | No | 12 |
| 20 |  | Vildagliptin/Metformin | 50mg/1000mg | Tabs | No | 5 |
| 21 |  | Metformin/Pioglitazone | 500mg/15mg | Tabs | No | 10 |
| 22 |  | Metformin/Rosiglitazone | 1000mg/2mg | Tabs | No | 10 |
| 23 |  | Glipizide/Metformin | 5mg/500mg | Tabs | No | 3 |
| 24 |  | Glimepiride/Pioglitazone | 4mg/15mg | Tabs | No | 3 |
| 25 |  | Metformin/Glibenclamide | 500mg/5mg | Tabs | No | 5 |
| 26 |  | Metformin/Glimepiride | 500mg/2mg | Tabs | No | 7 |
| 27 |  | Metformin/Glimepiride | 500mg/1mg | Tabs | No | 6 |
| 28 |  | Ertugliflozin/Sitagliptin | 15mg/100mg | Tabs | No | 1 |
| 29 |  | Empagliflozin/Linagliptin | 10mg/5mg | Tabs | No | 1 |
| 30 |  | Empagliflozin/Metformin | 10mg/1000mg | Tabs | No | 1 |

**Table S2:** Summary statistics of unit prices for oral antidiabetics by price category (LPG, HPG, OB) pre- and post-deregulation.

| ****Category**** | ****Period**** | ****Obs.**** | ****Mean****  ****(PKR)**** | ****Std. Dev.**** | ****Min.****  ****(PKR)**** | ****Max.****  ****(PKR)**** | ****Q1****  ****(PKR)**** | ****Q2****  ****Median-PKR**** | ****Q3****  ****(PKR)**** | ****IQR**** | ****Skewness**** | ****Kurtosis**** | Median (USD) | Median (EUR) |
| --- | --- | --- | --- | --- | --- | --- | --- | --- | --- | --- | --- | --- | --- | --- |
| LPG | Pre | 508 | 23.52 | 13.35 | 1.00 | 54.45 | 12.6 | 22.43 | 34.29 | 21.69 | 0.31 | 2.17 | 0.08 | 0.07 |
|  | Post | 520 | 26.89 | 19.86 | 1.00 | 78.57 | 13.81 | 25.71 | 37.36 | 23.55 | 6.24 | 90.02 | 0.09 | 0.09 |
| HPG | Pre | 231 | 31.55 | 13.58 | 3.43 | 54.45 | 24.00 | 35.00 | 40.00 | 16.00 | -0.36 | 2.27 | 0.13 | 0.12 |
|  | Post | 229 | 37.18 | 22.74 | 3.20 | 111.00 | 27.86 | 37.36 | 43.57 | 15.71 | 3.97 | 31.74 | 0.13 | 0.12 |
| OB | Pre | 259 | 33.17 | 27.63 | 3.56 | 110.70 | 8.62 | 21.00 | 41.00 | 32.38 | 0.79 | 2.61 | 0.08 | 0.07 |
|  | Post | 259 | 43.27 | 41.78 | 2.12 | 158.92 | 10.34 | 30.8 | 61.52 | 51.18 | 1.17 | 3.33 | 0.11 | 0.10 |

**Note**: 1 USD = 277.575 PKR; 1 EUR = 301.815 PKR (Source: <https://www.oanda.com/currency-converter/en/?from=EUR&to=PKR&amount=1> ).

**Table S3.** Median unit prices and statistical comparisons of oral antidiabetics by NEML status and formulation type before and after deregulation.

| **Pre/post deregulation** | **Product type** | **Grouping variable** | **Group** | **Median Unit Price** | **Observations** | **Rank Sum** | **Expected Rank Sum** | **z-value** | ***p*-value** |
| --- | --- | --- | --- | --- | --- | --- | --- | --- | --- |
| **Pre** | **LPG** | **NEML status** | non-NEML | 26.50 | 292 | 83689 | 74059.5 | 5.88 | 0.000 |
|  |  |  | NEML | 18.00 | 217 | 45597 | 55226.5 |  |  |
|  |  | **Formulation type** | Single-ingredient | 21.00 | 297 | 71173.5 | 75586.5 | -2.70 | 0.006 |
|  |  |  | Combination | 26.50 | 211 | 58112.5 | 53699.5 |  |  |
| **Post** | **LPG** | **NEML status** | non-NEML | 30.78 | 307 | 90281 | 79973.5 | 6.11 | 0.000 |
|  |  |  | NEML | 20.39 | 213 | 45179 | 55486.5 |  |  |
|  |  | **Formulation type** | Single-ingredient | 23.92 | 297 | 71486.5 | 76847.5 | -3.14 | 0.0016 |
|  |  |  | Combination | 31.79 | 225 | 63973.5 | 58612.5 |  |  |
| **Pre** | **HPG** | **NEML status** | non-NEML | 37.70 | 135 | 16389.5 | 14720 | 4.92 | 0.000 |
|  |  |  | NEML | 24.29 | 101 | 9945.5 | 11615 |  |  |
|  |  | **Formulation type** | Single-ingredient | 29.64 | 139 | 15509 | 16124 | -1.15 | 0.21 |
|  |  |  | Combination | 35.79 | 92 | 11287 | 10672 |  |  |
| **Post** | **HPG** | **NEML status** | non-NEML | 39.29 | 128 | 16389.5 | 14720 | 3.35 | 0.0008 |
|  |  |  | NEML | 35.00 | 101 | 9945.5 | 11615 |  |  |
|  |  | **Formulation type** | Single-ingredient | 35.00 | 139 | 15217.5 | 15985 | -1.56 | 0.1169 |
|  |  |  | Combination | 39.29 | 90 | 11117.5 | 10350 |  |  |
| **Pre** | **OB** | **NEML status** | non-NEML | 59.80 | 118 | 20043 | 15340 | 7.85 | 0.000 |
|  |  |  | NEML | 13.56 | 141 | 13627 | 18330 |  |  |
|  |  | **Formulation type** | Single-ingredient | 20.50 | 191 | 22650 | 24830 | -4.12 | 0.000 |
|  |  |  | Combination | 37.80 | 68 | 11020 | 8840 |  |  |
| **Post** |  | **NEML status** | non-NEML | 59.80 | 124 | 20240.5 | 16120 | 6.84 | 0.000 |
|  |  |  | NEML | 16.00 | 135 | 13429.5 | 17550 |  |  |
|  |  | **Formulation type** | Single-ingredient | 20.50 | 185 | 21700.5 | 24050 | -4.68 | 0.000 |
|  |  |  | Combination | 37.80 | 74 | 11969.5 | 9620 |  |  |

**Table S4.** **Linear regression analysis of the association between medicine unit prices and market competition (Number of registered brands) Pre- and Post-deregulation.**

| **Medicine Price Type** | **Pre/Post Deregulation** | **Coefficient (Registered Brands)** | **Std. Error** | ***t*-Value** | ***p*-Value** | **R-Squared** | **95% Confidence Interval** |
| --- | --- | --- | --- | --- | --- | --- | --- |
| LPG | Pre | -0.26 | 0.03 | -9.81 | <0.001 | 0.16 | [-0.308, -0.205] |
|  | Post | -0.20 | 0.04 | -4.74 | <0.001 | 0.04 | [-0.276, -0.114] |
| HPG | Pre | -0.33 | 0.05 | -7.04 | <0.001 | 0.18 | [-0.427, -0.240] |
|  | Post | -0.39 | 0.09 | -4.35 | <0.001 | 0.08 | [-0.568, -0.214] |
| OB | Pre | -0.52 | 0.06 | -8.16 | <0.001 | 0.21 | [-0.642, -0.392] |
|  | Post | -0.72 | 0.10 | -7.38 | <0.001 | 0.17 | [-0.912, -0.528] |

**Table S5.** Affordability of oral anti-diabetics pre and post price deregulation policy.

|  | | | | Pre-deregulation | | | Post-deregulation | | |
| --- | --- | --- | --- | --- | --- | --- | --- | --- | --- |
| Medicine | **ATC Code** | **DDD-WHO** | **No. of tabs** | **NDWs-LPG** | **NDW-HPG** | **NDW-OB** | **NDW-LPG** | **NDW-HPG** | **NDW-OB** |
| Sitagliptin 100mg Tabs | A10BH01 | 100mg | 1 | 1.40 | 1.47 | 3.12 | 1.50 | - | 3.12 |
| Vildagliptin 50mg Tabs | A10BH02 | 100mg | 2 | 1.35 | 1.71 | 4.46 | 1.61 | 2.14 | 7.58 |
| Repaglinide 2mg Tabs | A10BX02 | 4mg | 2 | 0.65 | - | 3.37 | 0.81 | - | 3.37 |
| Metformin 500mg Tabs | A10BA02 | 2000mg | 4 | 0.37 |  | 0.40 | 0.37 | 0.36 | 0.42 |
| Metformin 1g Tabs | A10BA03 | 2000mg | 2 | 0.20 | 0.23 | 0.35 | 0.23 | 0.23 | 0.40 |
| Empagliflozin 25mg Tabs | A10BK03 | 17.5mg | 1 | 0.97 | 1.13 | - | 0.90 | 1.26 | - |
| Empagliflozin 10mg Tabs | A10BK03 | 17.5mg | 2 | 1.37 | 1.37 | - | 1.76 | 1.97 | - |
| Dapagliflozin 5mg Tabs | A10BK01 | 10mg | 2 | 2.10 | 2.11 | - | 2.10 | 2.11 | - |
| Glipizide 5mg Tabs | A10BB07 | 10mg | 2 | 0.19 | 0.19 | - | 0.53 | 0.20 | - |
| Gliclazide 80mg Tabs | A10BB09 | 60mg | 1 | 0.37 | 0.38 | 0.38 | 0.60 | 0.72 | 0.38 |
| Glimepiride 1mg Tabs | A10BB12 | 2mg | 2 | 0.45 | - | 0.49 | 0.57 | - | 0.58 |
| Glimepiride 2mg Tabs | A10BB12 | 2mg | 1 | 0.38 | 0.41 | 0.58 | 0.42 | 0.57 | 0.58 |
| Glimepiride 3mg Tabs | A10BB12 | 2mg | 1 | 0.53 | 0.53 | 1.02 | 0.56 | 0.67 | 1.30 |
| Glimepiride 4mg Tabs | A10BB12 | 2mg | 1 | 0.56 | 0.79 | 1.15 | 0.81 | 1.04 | 1.15 |
| Glibenclamide 5mg Tabs | A10BB01 | 10mg | 2 | 0.18 | 0.19 | - | 0.19 | 0.23 | - |
| Pioglitazone 15mg Tabs | A10BG03 | 30mg | 2 | 1.20 | 1.67 | - | 1.45 | 1.67 | - |
| Rosiglitazone 4mg Tabs | A10BG02 | 6mg | 2 | 0.15 | - | - | 0.59 | - | - |
| metformin/sitagliptin 1g/50mg Tabs | A10BD07 | 1000mg/  50mg | 1 | 0.95 | 0.96 | 2.01 | 1.06 | 1.17 | 3.12 |
| metformin/sitagliptin 500mg/50mg Tabs | A10BD07 | 1000mg/  51mg | 2 | 1.92 | 2.11 | 4.02 | 1.94 | 2.31 | 5.45 |
| Vildagliptin/metformin 50mg/1g Tabs | A10BD08 | 1000mg/  50mg | 2 | 2.17 | 2.19 | 4.17 | 2.17 | 2.21 | 5.82 |
| Metformin/Pioglitazone 500mg/15mg Tabs | A10BD05 | 1000mg/  30mg | 2 | 0.79 | 0.79 | - | 0.80 | 0.88 | - |
| Metformin/Rosiglitazone 1g/2mg Tabs | A10BD03 | 1000mg/  6mg | 1 | 0.35 | 0.27 | - | 0.39 | 0.27 | - |
| Glipizide/Metformin 5mg/500mg Tabs | A10BB07 | 10mg/  1000mg | 2 | 0.71 | - | - | 0.78 | - | - |
| Glimepiride/Pioglitazone 4mg/15mg Tabs | A10BD06 | 2mg/  30mg | 1 | 0.58 | 0.53 | - | 1.10 | 1.23 | - |
| Metformin/Glibenclamide 500mg/5mg Tabs |  | up to 2000mg/  10mg | 2 | 0.32 | - | 0.32 | 0.37 | - | 0.45 |
| Metformin/Glimepiride 500mg/2mg Tabs |  |  | 2 | 0.99 | - | 2.13 | 1.06 | - | 2.13 |
| Metformin/Glimepiride 500mg/1mg Tabs |  |  | 2 | 0.59 | 0.59 | 1.18 | 0.68 | 0.72 | 1.18 |
| Ertugliflozin/Sitagliptin 15mg/100mg Tabs | A10BD24 | 10mg/  100mg | 1 | 1.29 | 1.29 | - | 1.40 | 1.56 | - |
| Empagliflozin/Linagliptin 10mg/5mg Tabs | A10BD19 | 17.5mg/  5mg | 1 | 0.87 | 1.06 | - | 0.95 | 1.17 | - |
| Metformin/ empagliflozin 1g/10mg Tabs | A10BD20 | 2000mg/  17.5mg | 1 | 0.75 | 0.75 | - | 0.90 | 1.02 | - |

**Table S6.** Percentage availability of oral anti-diabetics by NEML status and drug type.

| **Sr. no.** | **Medicine** | **NEML status** | **% Availability-Generics** | **% Availability-OB** | **Overall % availability** | **Availability category** |
| --- | --- | --- | --- | --- | --- | --- |
| 1 | Sitagliptin 100mg Tabs | 0 | 97 | 17 | 97 | High |
| 2 | Vildagliptin 50mg Tabs | 0 | 90 | 63 | 90 | High |
| 3 | Repaglinide 2mg Tabs | 0 | 17 | 40 | 40 | Low |
| 4 | Metformin 500mg Tabs | 1 | 63 | 97 | 97 | High |
| 5 | Metformin 1g Tabs | 0 | 17 | 47 | 47 | Fairly low |
| 6 | Empagliflozin 25mg Tabs | 1 | 90 | 0 | 90 | High |
| 7 | Empagliflozin 10mg Tabs | 1 | 83 | 0 | 83 | High |
| 8 | Dapagliflozin 5mg Tabs | 1 | 90 | 0 | 90 | High |
| 9 | Glipizide 5mg Tabs | 1 | 23 | 0 | 23 | Low |
| 10 | Gliclazide 80mg Tabs | 1 | 7 | 60 | 60 | Fairly high |
| 11 | Glimepiride 1mg Tabs | 1 | 83 | 70 | 83 | High |
| 12 | Glimepiride 2mg Tabs | 1 | 83 | 70 | 83 | High |
| 13 | Glimepiride 3mg Tabs | 1 | 83 | 70 | 83 | High |
| 14 | Glimepiride 4mg Tabs | 1 | 87 | 67 | 87 | High |
| 15 | Glibenclamide 5mg Tabs | 1 | 47 | 0 | 47 | Fairly low |
| 16 | Pioglitazone 15mg Tabs | 0 | 63 | 0 | 63 | Fairly high |
| 17 | Rosiglitazone 4mg Tabs | 0 | 7 | 3 | 7 | Low |
| 18 | metformin/sitagliptin 1g/50mg Tabs | 0 | 90 | 27 | 90 | High |
| 19 | metformin/sitagliptin 500mg/50mg Tabs | 0 | 93 | 27 | 93 | High |
| 20 | Vildagliptin/metformin 50mg/1g Tabs | 0 | 73 | 50 | 73 | Fairly high |
| 21 | Metformin/Pioglitazone 500mg/15mg Tabs | 0 | 73 | 0 | 73 | Fairly high |
| 22 | Metformin/Rosiglitazone 1g/2mg Tabs | 0 | 7 | 0 | 7 | Low |
| 23 | Glipizide/Metformin 5mg/500mg Tabs | 0 | 7 | 0 | 7 | Low |
| 24 | Glimepiride/Pioglitazone 4mg/15mg Tabs | 0 | 43 | 0 | 43 | Low |
| 25 | Metformin/Glibenclamide 500mg/5mg Tabs | 0 | 23 | 37 | 37 | Low |
| 26 | Metformin/Glimepiride 500mg/2mg Tabs | 0 | 93 | 57 | 93 | High |
| 27 | Metformin/Glimepiride 500mg/1mg Tabs | 0 | 80 | 50 | 80 | High |
| 28 | Ertugliflozin/Sitagliptin 15mg/100mg Tabs | 0 | 47 | 0 | 47 | Fairly low |
| 29 | Empagliflozin/Linagliptin 10mg/5mg Tabs | 0 | 77 | 0 | 77 | Fairly high |
| 30 | Metformin/ empagliflozin 1g/10mg Tabs | 0 | 53 | 0 | 53 | Fairly low |
